# *In Vivo* Delta Opioid Receptor Internalization Controls Behavioral Effects of Agonists

**DOI:** 10.1371/journal.pone.0005425

**Published:** 2009-05-01

**Authors:** Amynah A. A. Pradhan, Jérôme A. J. Becker, Grégory Scherrer, Petra Tryoen-Toth, Dominique Filliol, Audrey Matifas, Dominique Massotte, Claire Gavériaux-Ruff, Brigitte L. Kieffer

**Affiliations:** Institut de Génétique et de Biologie Moléculaire et Cellulaire, Centre National de la Recherche Scientifique/Institut National de la Santé et de la Recherche Médicale/Université Louis Pasteur, Illkirch, France; University of California, Berkeley, United States of America

## Abstract

**Background:**

GPCRs regulate a remarkable diversity of biological functions, and are thus often targeted for drug therapies. Stimulation of a GPCR by an extracellular ligand triggers receptor signaling via G proteins, and this process is highly regulated. Receptor activation is typically accompanied by desensitization of receptor signaling, a complex feedback regulatory process of which receptor internalization is postulated as a key event. The *in vivo* significance of GPCR internalization is poorly understood. In fact, the majority of studies have been performed in transfected cell systems, which do not adequately model physiological environments and the complexity of integrated responses observed in the whole animal.

**Methods and Findings:**

In this study, we used knock-in mice expressing functional fluorescent delta opioid receptors (DOR-eGFP) in place of the native receptor to correlate receptor localization in neurons with behavioral responses. We analyzed the pain-relieving effects of two delta receptor agonists with similar signaling potencies and efficacies, but distinct internalizing properties. An initial treatment with the high (SNC80) or low (AR-M100390) internalizing agonist equally reduced CFA-induced inflammatory pain. However, subsequent drug treatment produced highly distinct responses. Animals initially treated with SNC80 showed no analgesic response to a second dose of either delta receptor agonist. Concomitant receptor internalization and G-protein uncoupling were observed throughout the nervous system. This loss of function was temporary, since full DOR-eGFP receptor responses were restored 24 hours after SNC80 administration. In contrast, treatment with AR-M100390 resulted in retained analgesic response to a subsequent agonist injection, and *ex vivo* analysis showed that DOR-eGFP receptor remained G protein-coupled on the cell surface. Finally SNC80 but not AR-M100390 produced DOR-eGFP phosphorylation, suggesting that the two agonists produce distinct active receptor conformations *in vivo* which likely lead to differential receptor trafficking.

**Conclusions:**

Together our data show that delta agonists retain full analgesic efficacy when receptors remain on the cell surface. In contrast, delta agonist-induced analgesia is abolished following receptor internalization, and complete behavioral desensitization is observed. Overall these results establish that, in the context of pain control, receptor localization fully controls receptor function *in vivo*. This finding has both fundamental and therapeutic implications for slow-recycling GPCRs.

## Introduction

G protein coupled receptors (GPCRs) form the largest family of membrane receptors [1]. A variety of physiological functions are regulated by GPCRs, which represent the most common target for therapeutic drugs. Stimulation of a GPCR by an extracellular messenger, either physiological or synthetic, triggers intracellular receptor signaling via heterotrimeric G proteins. This process is highly regulated and receptor activation is typically accompanied by desensitization of receptor signaling, a complex feedback regulatory process whereby receptor responsiveness decreases upon continued agonist stimulation. Receptor trafficking is considered to be a key process in the regulation of receptor signaling. In particular, many studies have shown that receptor stimulation by an agonist concomitantly leads to receptor signaling and redistribution of receptor molecules away from the cell surface (for review see [2]–[3]).

The significance of receptor endocytosis in the regulation of GPCR function is under intense investigation, and many aspects deserve further clarification. First, receptor internalization may influence agonist efficacy in different ways. The most straightforward hypothesis proposes that receptor internalization reduces agonist effects, as fewer receptors are available at the cell surface. On the contrary, it has also been suggested that receptor endocytosis promotes rapid resensitization by recycling the receptor back to the cell surface, which contributes to the maintenance of a large population of active receptors at the plasma membrane [4]. Second, receptor internalization may not simply terminate intracellular signaling. Classically, agonist binding results in phosphorylation of the GPCR, which in turn leads to recruitment of β-arrestins. Binding to these adaptor proteins initiates receptor internalization and physically prevents further receptor interaction with heterotrimeric G proteins. However, recent findings suggest that the internalized receptor-β-arrestin complex can in turn recruit signaling proteins and initiate further intracellular signaling [1], [5], [6]. Third, the physiological relevance of many receptor trafficking studies is limited, as the majority have been performed in transfected cellular models. These *in vitro* systems may not reflect *in vivo* situations in terms of receptor density, protein content of receptor-expressing cells, or even receptor localization within subcellular compartments, as is the case for neurons [3]. Additionally, data from cellular models provide no understanding of how receptor trafficking influences integrated responses in the living organism. Fourth, individual GPCRs vastly differ in their trafficking properties, leading to specific regulatory mechanisms for each receptor. Overall, the characterization of receptor trafficking in native tissues is only beginning [7].

Due to limited availability of specific antibodies, *in vivo* trafficking of GPCRs has been investigated for only a limited number of native receptors [8]–[12]. Recently we have created knock-in mice expressing a fully functional fluorescent delta opioid receptor (DOR-eGFP) in place of the endogenous delta receptor. In these animals DOR-eGFP are expressed at physiological levels within their native environment. Furthermore, these receptors are directly visible *in vivo*. These mutant mice have proven to be an exceptional tool in studying receptor neuroanatomy, real-time receptor trafficking in live neurons, and receptor movements *in vivo* [13]. This unique animal model can now be used to determine the relationship between receptor trafficking in neurons and receptor function at a behavioral level. Our previous work using DOR-eGFP mice showed that treatment with the delta agonist SNC80 triggered massive receptor endocytosis throughout the nervous system, together with locomotor activation. We further showed that mice with internalized receptors did not show locomotor activation following a second drug administration [13]. This was a first indication that internalization may impact delta receptor function *in vivo*, at least in the case of locomotor responses.

The opioid system is involved in pain control, reward processing, and stress responses. Genetic approaches have revealed that the delta receptor fulfills roles highly distinct from those of mu and kappa opioid receptors [14], [15]. Several studies have shown that delta receptors can specifically alleviate persistent pain [16]–[20]. In the present study we examine the regulation of delta opioid receptor function in the control of inflammatory pain. We first characterize trafficking properties of two delta receptor agonists in live neurons from DOR-eGFP mice. We show that the two compounds have very distinct internalizing properties, despite similar *in vitro* signaling potencies and efficacies. Further, we find *in vivo* that a first injection of each agonist in DOR-eGFP mice reduces inflammatory pain, with similar efficacy for the two drugs. Importantly, we find that a subsequent agonist administration *in vivo* has very distinct consequences on the behavioral response. The high-internalizing agonist no longer relieves pain, indicating that acute *in vivo* desensitization has occurred. In contrast the low-internalizing agonist remains fully active following the second administration, demonstrating that non-internalized receptors remain functional. Finally, we show that receptor phosphorylation and uncoupling parallels receptor internalization, and that restoration of surface receptors reinstate opioid analgesia. These data unambiguously demonstrate that receptor internalization fully determines drug efficacy *in vivo*.

## Results

### SNC80 and ARM390 show similar pharmacology at the DOR-eGFP receptor

SNC80 [21] is a widely used non-peptidic compound that shows high delta selectivity *in vivo*, and was chosen as a reference delta receptor agonist in this study. AR-M100390 (ARM390) is a close SNC80 derivative [22], reported to be a poorly internalizing agonist in a neuroblastoma cell line [23]. We compared ARM390 and SNC80 activities throughout this study. Met-enkephalin was also examined *ex vivo*, as a prototypic endogenous delta opioid receptor agonist.

We first characterized the pharmacological profiles of the three delta receptor agonists in brain membranes prepared from DOR-eGFP mice. In competition binding experiments, all three ligands displaced [^3^H]naltrindole with binding affinities in the nanomolar range (Table 1). The two synthetic alkaloids, SNC80 and ARM390, had affinities which were approximately 10 times greater than the affinity of Met-enkephalin. We compared functional responses of all three ligands in the [^35^S]GTPγS binding assay (Table 1). The three agonists had similar potencies. Both SNC80 and ARM390 produced similar levels of receptor stimulation, while Met-enkephalin was slightly more efficacious. SNC80 and ARM390 therefore bind to and activate the DOR-eGFP receptor with comparable potencies and efficacies.

**Table 1:** Pharmacological and internalization properties of delta agonists at the DOR-eGFP receptor (see Materials and Methods).

	Affinity (Competition Binding)	G protein coupling ([^35^S]GTPγS binding)	
Ligand	Ki (nM)	EC_50_ (nM)	E_max_ (% basal)
SNC80	9.1±0.5***	121.5±36.3	228.4±8.7
ARM390	2.3±0.3***	169.3±9.5	214.3±9.6
Met-enkephalin	66.85±12.9	94.57±4.0	294.2±3.9***

Endocytosis efficiencies			
	Concentration (nM)	Striatum	Hippocampus
		E_1/2_ (min)	E_1/2_ (min)
SNC80	1000	1.8±0.5	3.3±0.8
	100	3.8±0.8	4.9±0.5
	10	10.6±1.8	14.9±2.0
ARM390	1000	4.7±0.7	5.8±0.3
	100	ND	ND
Met-enkephalin	1000	1.32±0.22	1.41±0.37
	100	3.93±0.16	2.87±0.52
	10	7.13±0.98	4.06±0.75

For competition binding experiments, affinities are shown as Ki values. For the [^35^S]GTPγS assay, ligand potencies are expressed as EC_50_ values, and maximum activation levels are indicated as E_max_. Basal binding (100%) is defined as [^35^S]GTPγS responses in absence of ligand. All data are expressed as mean±SEM from 3–4 independent experiments performed in duplicate with two different membrane preparations.

*** p<0.001, one-way ANOVA. In primary neuron cultures, quantification of agonist-induced internalization was performed using real time confocal microscopy. The number of DOR-eGFP vesicles at various time points was counted from the corresponding videos. When internalization occurred it was completed by 60 min, and the number of DOR-eGFP vesicles at this time point was defined as 100%. Endocytosis efficiencies (E_1/2_) were defined as the time needed to internalize 50% of DOR-eGFP. ND indicates that weak or no change in surface labeling was detected. Data are mean±SEM for 4–9 independent experiments.

### SNC80 and ARM390 differentially internalize the DOR-eGFP receptor in live neurons

**Figure 1:**
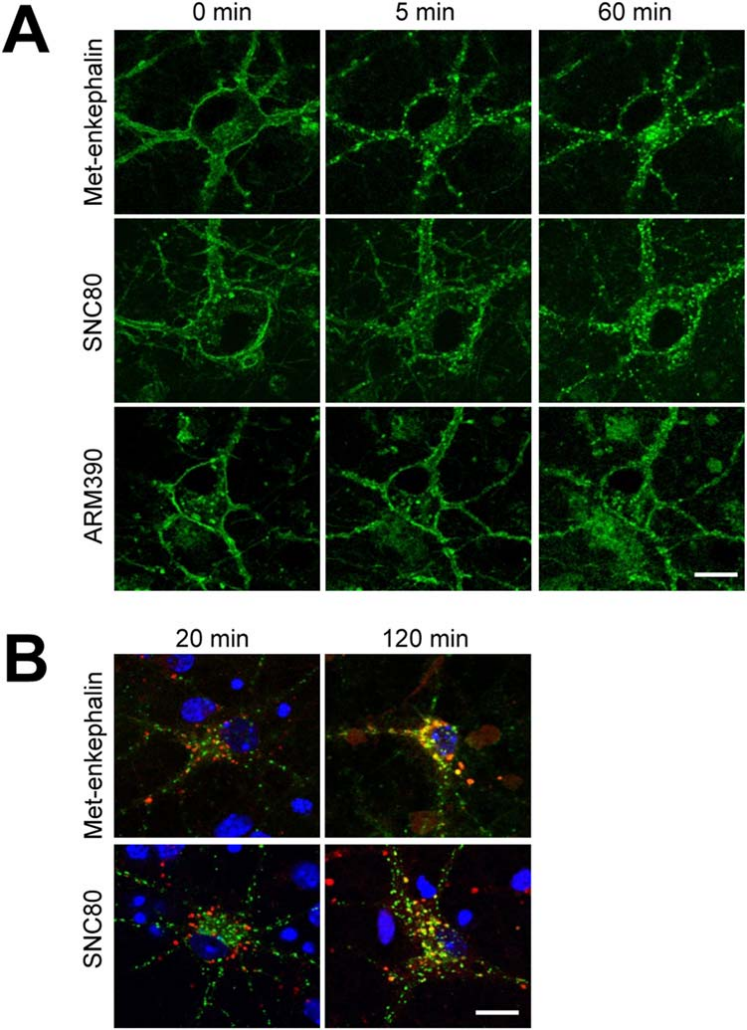
Delta agonists differentially internalize DOR-eGFP in primary neurons. Striatal and hippocampal primary neurons were treated for 60 minutes with Met-enkephalin, SNC80 or ARM390. (A) Representative images of hippocampal neurons treated with 100 nM of agonists are shown. Scale bar is 12 µm. (B) Internalized DOR-eGFP co-localized with lysosomes. Striatal primary neurons were incubated with Met-enkephalin (100 nM) or SNC80 (100 nM) for 20 or 120 minutes, along with LysoTracker Red DND 99 (300 nM); scale bar is 12 µm.

Data mainly from transfected cellular models ([24], and references therein), and also from *in vivo* experiments [12], [25], [26], indicate that delta agonists trigger delta receptor internalization. Similarly, our initial examination of primary neurons from DOR-eGFP mice, showed that Met-enkephalin and SNC80 trigger rapid internalization of the fluorescent receptor [13]. Here we compared internalization evoked by Met-enkephalin, SNC80 and ARM390 in live hippocampal and striatal neurons from DOR-eGFP mice, by real time confocal microscopy. Prior to drug administration, DOR-eGFP fluorescence was distributed along the entire cell membrane. At all tested concentrations (10 nM, 100 nM, 1 µM) Met-enkephalin and SNC80, induced rapid DOR-eGFP cluster formation and complete loss of surface labeling (see Figure 1A and Movies S1 and S2). In addition, co-expression of these internalized receptors with a lysosome marker (LysoTracker Red DND 99; Figure 1B) indicated that DOR-eGFP receptors were targeted to the lysosomal compartment. In contrast, 100 nM ARM390 failed to induced receptor endocytosis (Figure 1A and Movie S3). A 100-times higher ARM390 concentration was required to internalize DOR-eGFP (Movie S4). Similar results were obtained for both neuronal preparations (Table 1).

In conclusion, although SNC80 and ARM390 having similar binding properties, the two drugs produce distinct internalization efficacies at DOR-eGFP receptors. SNC80 is a high-internalizing agonist whereas ARM390 appears to be a low-internalizing agonist under *ex vivo* experimental conditions.

### SNC80 and ARM390 differentially regulate DOR-eGFP receptor function *in vivo*

We next determined whether equipotent (see Figure S1) doses of SNC80 and ARM390 differentially affected behavior in a model of inflammatory pain. DOR-eGFP mice were tested in the Complete Freud's Adjuvant (CFA) model of inflammatory pain [27]. In this behavioral model, delta agonists show anti-allodynic and anti-hyperalgesic properties [17]–[19] and delta receptor knockout mice display enhanced pain [28]. CFA was injected either in the paw or the tail of DOR-eGFP mice to measure mechanical or thermal responses, respectively.

**Figure 2:**
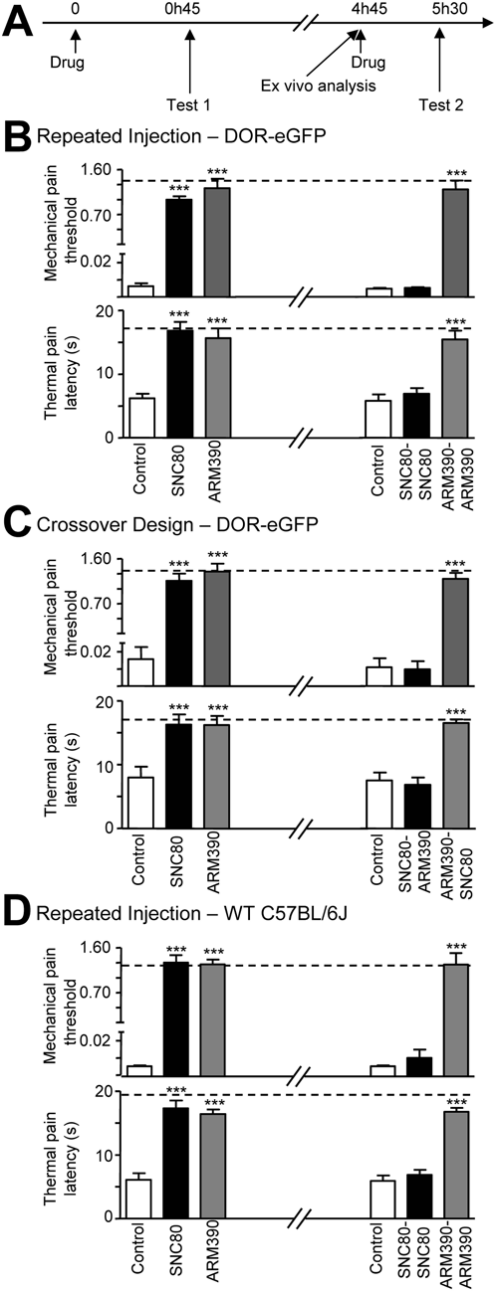
Differential *in vivo* regulation of DOR by SNC80 and ARM390. (A) Time line of the experiments is shown on top. (B, C and D) Test 1: mechanical (CFA paw) and thermal (CFA tail) responses in animals treated with vehicle (Control), SNC80 (10 mg/kg) or ARM390 (10 mg/kg). Test 2: animals re-challenged four hours later with the same drug (B and D) or the other drug (C). Dashed lines represent baseline mechanical or thermal responses pre-CFA. For drug effects *** p<0.001, two-way RM ANOVA, n = 5–8 mice/group.

Forty-eight hours post-CFA, we observed mechanical allodynia using von Frey stimulation (paw CFA) and heat hyperalgesia in a tail immersion assay (tail CFA) (Figure 2B, C and D, dashed line *vs.* control). CFA-evoked pain was almost completely abolished with the first administration of either SNC80 or ARM390, and both produced comparable effects (Figure 2B, C and D, Test 1). However, a subsequent injection of SNC80 or ARM390 produced distinct responses. The pain-relieving effects of SNC80 were completely lost while ARM390 remained fully active (Figure 2B, Test 2).

To determine if the acute behavioral desensitization after SNC80 treatment could be generalized to other delta agonists, we performed a cross-over experiment (Figure 2C). As before, the first injection of SNC80 and ARM390 similarly reversed both CFA-induced mechanical and thermal pain (Test 1). Mice treated with SNC80 were subsequently administered ARM390, but this injection was ineffective. Conversely animals treated with ARM390 were re-challenged with SNC80, and this significantly attenuated both mechanical and thermal pain responding (Figure 2C, Test 2).

In order to address the possibility that these results were limited to DOR-eGFP mice, we tested for acute behavioral desensitization in commercial C57BL/6J mice (Figure 2D). The results were similar to those obtained with DOR-eGFP mice. CFA in the paw or tail produced robust allodynic or hyperalgesic responses, which were completely reversed by the first injection of SNC80 or ARM390 (Figure 2D, Test 1). As seen previously, a subsequent injection of SNC80 was ineffective, but repeated injection of ARM390 continued to attenuate mechanical allodynia and thermal hyperalgesia (Figure 2D, Test 2).

Altogether, SNC80 treatment prevents further responding to either agonist, whereas ARM390 treatment does not disrupt subsequent responses to the two drugs. Therefore, initial exposure to the high-internalizing but not the low-internalizing agonist abolishes DOR function *in vivo*.

### DOR-eGFP receptor internalization *in vivo* parallels receptor phosphorylation and uncoupling from G proteins

We characterized the status of DOR-eGFP receptors in neurons, at the time of the second injection. Three other groups of animals were treated identically to control, SNC80 and ARM390 groups, but were sacrificed for *ex vivo* analysis, instead of receiving the second drug treatment (see time line in Figure 2).

**Figure 3:**
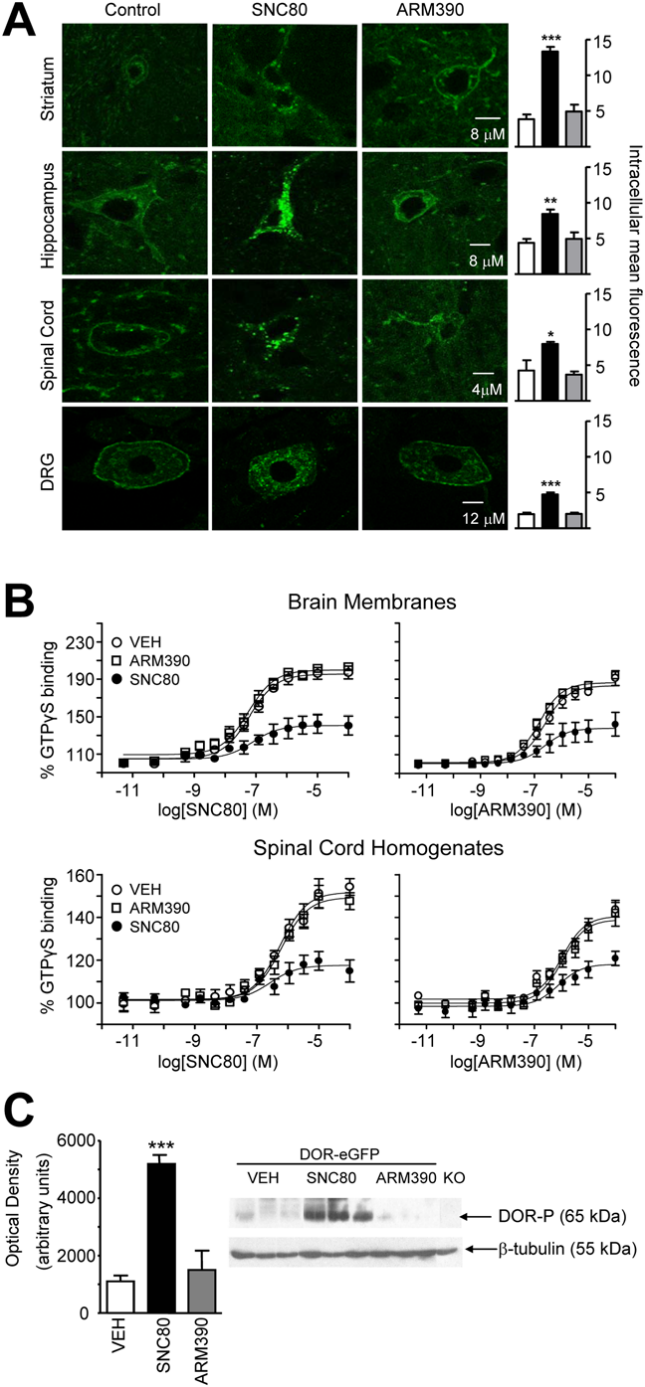
SNC80, but not ARM390, triggers DOR-eGFP internalization, uncoupling, and phosphorylation *in vivo*. Separate groups of DOR-eGFP mice were treated as in Figure 2, but instead of the second drug administration (A and B) or 20 min later (C) tissue was harvested for *ex vivo* analysis. (A) CNS regions and dorsal root ganglia were analyzed by confocal microscopy and representative images are shown. Mean intracellular DOR-eGFP fluorescence was quantified in 5–7 sections/region/mouse. White bars, control group; black bars, SNC80 group; grey bars, ARM390 group; *p<0.05, **p<0.01, *** p<0.001, one-way ANOVA, n = 3–5 mice/group. (B) [^35^S]GTPγS concentration-response curves to SNC80 and ARM390. y-axis shows mean±SEM specific [^35^S]GTPγS binding expressed as percentage basal binding (i.e. absence of agonist). Experiments were performed in triplicate; n = 3–5 mice/group. (C) Western blot of phospho-DOR (Ser 363) in hippocampal samples collected 20 min post-drug injection. KO, DOR knockout mouse challenged with SNC80 (10 mg/kg). Mean optical density was assessed for n = 3 mice/group.

In the first group of animals we examined DOR-eGFP subcellular localization in three CNS regions (striatum, hippocampus, and spinal cord) as well as in dorsal root ganglia using confocal microscopy (Figure 3A). SNC80-treated animals showed robust internalization of DOR-eGFP in all regions examined. Even almost 5 hours after drug administration, very little DOR-eGFP was observed on the cell surface of neurons, in both cell bodies and processes. In contrast, almost no DOR-eGFP vesicles were observed in the ARM390 group, where continuous fluorescent labeling was clearly located on the cell surface. Quantification of intracellular fluorescence confirmed that SNC80, but not ARM390, induced internalization (Figure 3A, histogram). Noticeably, injections of higher doses of ARM390 (30 and 60 mg/kg) also failed to produce DOR-eGFP internalization (data not shown), confirming the poor internalization potency of this compound *in vivo*. Hence, although both agonists showed similar pain relieving properties, only SNC80 produced DOR-eGFP internalization *in vivo* in both central and peripheral nervous systems.

In the second group of animals, we investigated DOR-eGFP coupling to G proteins in brain membranes and spinal cord homogenates at the time of the second injection (Figure 3B). Concentration response curves in the [^35^S]GTPγS binding assay were established to both SNC80 and ARM390. SNC80-treated animals showed a 50–70% decrease in E_max_ responses, indicative of cellular desensitization. However, the ARM390-treated group showed similar [^35^S]GTPγS binding to the control group, demonstrating that surface receptors remained functionally coupled.

In the third group, we determined whether SNC80 and ARM390 induce different DOR-eGFP phosphorylation states, as GPCR desensitization is often preceded by agonist-induced receptor phosphorylation [7], [29]. DOR-eGFP mice were treated with drug, and the presence of phospho-DOR Ser(363) was determined using western blot (Figure 3C). Only SNC80 treatment produced significantly higher levels of phosphorylated receptor as compared to controls. To control for the specificity of the antibody, delta opioid receptor knockout mice were also treated with SNC80 (10 mg/kg ), and in this case no phospho-DOR band was detected. Thus, SNC80-induced phosphorylation of DOR-eGFP likely contributes to receptor desensitization, a phenomenon not observed after ARM390 treatment.

Hence, SNC80 triggered DOR-eGFP phosphorylation, uncoupling and endocytosis, while none of these events occurred with ARM390. This result indicates that regulatory processes, which occur at the neuronal level, are triggered by SNC80 only and are likely associated *in vivo*.

### Behavioral desensitization is transient

**Figure 4:**
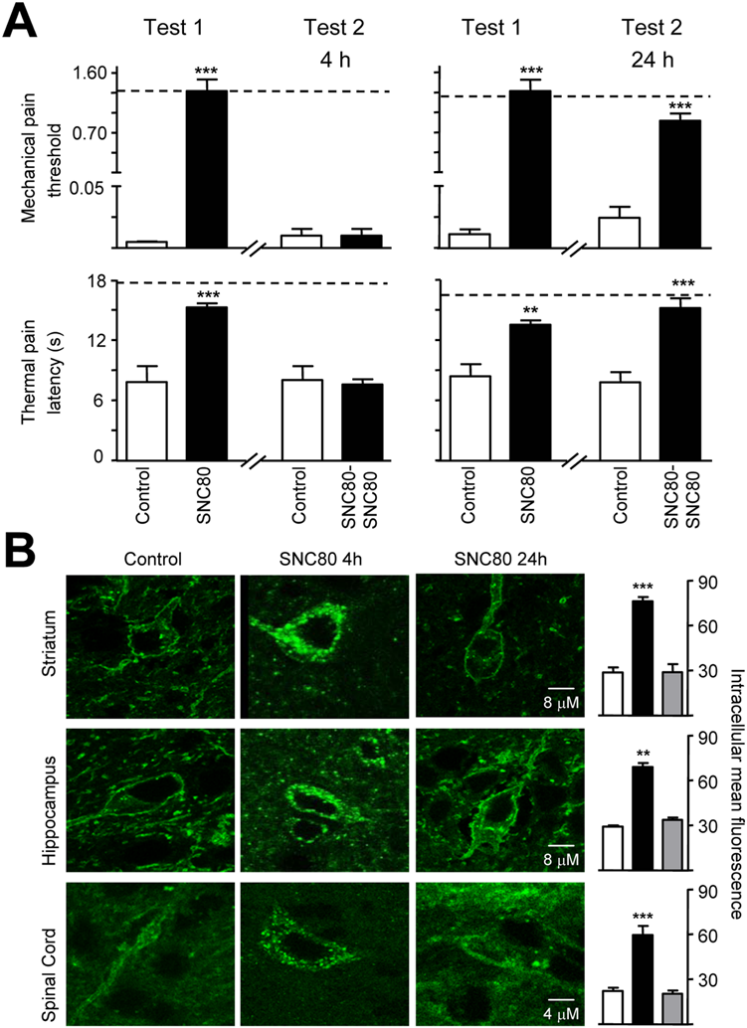
Restoration of DOR-eGFP function. (A)Test 1: mechanical (CFA paw) and thermal (CFA tail) responses in animals treated with vehicle (Control) or SNC80 (10 mg/kg). Test 2: animals re-challenged 4 (left panels) or 24 hours (right panels) with SNC80. Dashed lines represent baseline mechanical or thermal responses pre-CFA. For drug effects * p<0.05, **p<0.01, two-way RM ANOVA, n = 4–8 mice/group. (B) CNS regions were analyzed by confocal microscopy, and representative images are shown. Mean intracellular DOR-eGFP fluorescence was quantified in 5–7 sections/region/mouse. White bars, control group; black bars, SNC80 group re-challenged 4 hours after Test 1; grey bars, SNC80 group re-challenged 24 hours after Test 1; **p<0.01, *** p<0.001, one-way ANOVA, n = 3–5 mice/group.

We finally determined whether SNC80-induced behavioral desensitization could be reversed over time (Figure 4A). As seen previously, first exposure to SNC80 significantly attenuated allodynia, and a subsequent injection 4 hours later was ineffective. In contrast, mice that were re-challenged 24 hours following the first injection showed a clear anti-allodynic response to SNC80. Correspondingly, robust DOR-eGFP endocytosis was observed 4, but not 24 hours following drug treatment (Figure 4B). Hence, SNC80-induced internalization and the concomitant behavioral desensitization are transient phenomena.

## Discussion

### Delta opioid receptors undergo long-term sequestration *in vivo*

GPCR trafficking to and from the cell surface has been extensively studied in cellular models [2]. Agonist-induced endocytosis reduces the number of receptors accessible to extracellular agonists, and decreases drug efficacy shortly after internalization. Subsequently, internalized GPCRs can be sorted into multiple regulatory pathways. Some receptors recycle rapidly from early endosomes, leading to prompt resensitization of receptor function. Other receptors are targeted to late endosomes and may recycle slowly or be targeted for lysosomal degradation, resulting in prolonged attenuation of agonist-induced responses (for review see [2], [5]).

Many studies in neuronal and non-neuronal cell lines indicate that delta receptors are degraded after agonist-induced internalization [30]–[33]. However, no study has addressed the trafficking of delta receptors *in vivo*. Our data indicate that receptor internalization does not result in fast receptor recycling in DOR-eGFP mice. Four hours after the first injection of SNC80, a substantial amount of DOR-eGFP remained in intracellular clusters, and surface fluorescence was undetectable in neurons from all areas examined. One cannot exclude the possibility that some recycling of DOR-eGFP occurred after SNC80 treatment, which was not detectable in our experimental conditions. However, our behavioral data indicate that at this time point there was not enough DOR-eGFP on the cell surface to respond to the second infusion of agonist. Therefore, the predominant effect of agonist-induced internalization was long-term sequestration. Furthermore, in primary neurons from DOR-eGFP mice treated with SNC80 or Met-enkephalin, we found that internalized DOR-eGFP colocalized with a lysosomal marker as soon as two hours after agonist exposure. These data strongly suggest that internalized delta opioid receptors are targeted for degradation in living neurons. Together, our *ex vivo* and *in vivo* data definitively classify delta receptors among slow-recycling/degrading GPCRs. It remains to be determined if the restored pool of functional DOR-eGFP receptors observed at the surface of neurons 24 hours after agonist treatment arises from slow externalization of internal receptor pools or receptor neosynthesis.

### Distinct ligand-dependent conformational states control DOR-eGFP activity *in vivo*

The results of this study indicate that DOR-eGFP internalization was strongly correlated to receptor phosphorylation and uncoupling from G proteins. SNC80 produced robust DOR-eGFP endocytosis, which was concomitant with an increase in receptor phosphorylation and a decrease in [^35^S]GTPγS binding. ARM390 did not produce internalization, and correspondingly there was no change in [^35^S]GTPγS responses. Thus, in our experiments receptor coupling and internalization either occurred together or not at all. Evidence from previous *in vitro* studies suggest that GPCR uncoupling can occur without internalization. In neuroblastoma cells, ARM390 incubation resulted in desensitization of the cAMP response, but no change in receptor internalization [23]. In the same cell line, pharmacological treatments that block receptor internalization, such as concanavalin A and hypertonic sucrose, did not affect receptor desensitization [34]. In this study, we observed no dissociation between these two events *in vivo*, suggesting that in this case *in vitro* observations may not always predict *in vivo* processes.

One may speculate on molecular mechanisms governing the *in vivo* phosphorylation-uncoupling-internalization regulatory response that we observed. Studies in transfected systems have proposed a general scheme for the desensitization of GPCR signaling. The agonist-activated receptor is initially phosphorylated by GPCR kinases (GRKs), and the phosphorylated receptor, in turn, recruits β-arrestin to the cell surface [6]. Arrestins promote receptor internalization, and also physically prevent further receptor coupling to G proteins (for review see [2], [29]). Considering this scenario, we observed that SNC80 but not ARM390 produced DOR-eGFP phosphorylation on the Ser363 site. This site is a primary phosphorylation site following delta agonist stimulation, and plays a significant role in subsequent delta receptor desensitization and internalization [35], [36]. The difference in the ability of SNC80 and ARM390 to produce phosphorylation at this site, likely accounts for the divergent internalization profiles of these two drugs. Further, a recent study in transfected cells showed that delta receptors can adopt several ligand-specific conformations, which produce different G protein signaling complexes [37]. In our study, both SNC80 and ARM390 clearly activate a signaling cascade that leads to pain inhibition. However, ARM390 may bind in a mode that produces receptor signaling, but may not cause subsequent receptor phosphorylation and internalization. In contrast, SNC80 may interact differentially with the receptor binding pocket to promote a distinct active conformation, which in turn triggers signaling, as well as receptor phosphorylation and internalization. Consistent with this hypothesis of two different receptor-agonist complexes, molecular models that compared the two ligands indicated that both compounds bound to the same region of the delta receptor, but that ARM390 only partially covered the conformational space occupied by SNC80 [22].

### A therapeutic potential for non-internalizing delta agonists?

In this study we extensively characterized the *in vitro* and *in vivo* properties of ARM390. This compound is a derivative of SNC80, and has a higher selectivity for delta over mu and kappa opioid receptors [22]. In DOR-eGFP brain membranes, ARM390 acted as an agonist and had binding affinities and potencies similar to SNC80, consistent with the initial characterization of the compound in transfected cells [22]. Further, ARM390 did not produce substantial receptor internalization *in vivo*, as previously observed in cell lines [23]. The lack of ARM390-induced internalization correlated with continued receptor coupling to G proteins and *in vivo* efficacy of ARM390 at the second drug administration. The inability of ARM390 to induce receptor endocytosis, therefore, maintained full receptor function across two sequential drug treatments. From a therapeutic perspective, these properties could be advantageous for chronic treatments. Previous studies have shown the development of rapid tolerance to some of the behavioral effects of SNC80 in rats [38]. Further, tolerance to the effects of SNC80 on food response rates was also observed in rhesus monkeys following both acute and chronic exposure to the drug [39]. A non-internalizing delta agonist may induce less analgesic tolerance, although this remains to be studied in chronic treatment paradigms.

More generally, molecular mechanisms underlying tolerance are likely unique to each GPCR-agonist combination. To date the relationship between receptor internalization and *in vivo* tolerance has largely been debated in the context of the mu opioid receptor, where morphine tolerance represents a major clinical limitation (see [40]). A classical view is that mu receptor internalization strongly contributes to tolerance, supported notably by the absence of tolerance to analgesic effects of morphine in β-arrestin 2 knockout animals [41]. The observations that morphine poorly internalizes mu receptors [9], [10], [42], and that sequestered mu receptors resensitize rapidly by recycling ([40] and references therein), has led to a different theory of opioid tolerance. Potent internalizing mu agonists would allow continuous interruption and subsequent restoration of receptor function, whereas morphine would produce accelerated *in vivo* tolerance via adaptive cellular responses to uninterrupted signaling [43], [44]. However, mechanistic hypotheses from mu receptor data remain speculative, and are not directly transposable to delta receptors where rapid recycling does not occur.

In conclusion, we establish for the first time a direct link between the localization and function of the delta opioid receptor *in vivo*. Delta agonists reduce inflammatory pain, and the lack of receptor internalization maintains the response whereas receptor endocytosis acutely desensitizes this response. Our approach may be valuably extended to other slow-recycling GPCRs, and increase our knowledge of regulatory mechanisms driving *in vivo* GPCR function. The application of these findings may also have important consequences for drug discovery in many therapeutic areas.

## Materials and Methods

### DOR-eGFP mice

All experiments were performed in accordance with the European Communities Council Directive of 24 November 1986. Knock-in mice were produced by homologous recombination. In these mice the eGFP cDNA was introduced into exon 3 of the delta opioid receptor gene, in frame and 5′ from the stop codon [13]. Receptor binding and signaling properties were unchanged in DOR-eGFP mutants. Mice aged 12 weeks on average, were housed in a temperature- and humidity-controlled animal colony on a 12 h dark-light cycle. Food and water were available *ad libitum*.

### Delta agonists

SNC80 [21] is a widely used non-peptidic compound that shows high delta selectivity *in vivo*, and was selected as a reference delta agonist in this study (Tocris). AR-M100390 (ARM390, N,N-diethyl-4-(phenyl-piperidin-4-ylidenemethyl)-benzamide) is a SNC80 derivative [22], reported to be a poorly internalizing agonist in a neuroblastoma cell line [23] and was synthesized at AstraZeneca R&D Montreal (Canada). ARM390 was administered *per os* (p.o) by gavage, as it is an irritant when injected i.p. (AstraZeneca personal communication).

### *Ex vivo* tissue analysis of DOR-eGFP mice

Membrane preparations were carried out as described previously [45]. Whole brain and the lumbar segment of the spinal cord were removed, immediately frozen in isopentane or dry ice, and stored at −80°C prior to use. For the brain, [^3^H]naltrindole and [^35^S]GTPγS assays were performed on whole brain membranes. The [^35^S]GTPγS assay for the spinal cord was performed on homogenates. Whole brain membranes were prepared by homogenizing the brain in ice-cold 0.25 M sucrose solution 10 vol (ml/g wet weight of tissue). Samples were then centrifuged at 1100 g for 10 min. Supernatants were collected and diluted 5 times in buffer containing 50 mM TrisHCl (pH 7.4) and 1 mM EDTA, following which they were centrifuged at 35 000 g for 30 min. The pellets were homogenized in 2 ml ice-cold sucrose solution (0.32 M), aliquoted and kept at −80°C until further use. Spinal cords were prepared for binding by homogenization in the binding buffer (50 mM TrisHCl, 3 mM MgCl_2_, 0.2 mM EGTA, 100 mM NaCl, pH7.4) and used immediately.

For competition studies, 50 µg of membrane proteins were incubated with 1 nM [^3^H]naltrindole, in the presence of variable concentrations (10^−4^ to 10^−12^ M) of SNC80 or ARM390 for 1 h at 25°C. Membranes were washed and filtered, and radioactivity was quantified using a liquid scintillation counter. Assays were performed in duplicates, in 3 to 4 experiments using 2 different membrane preparations.

For each [^35^S]GTPγS binding assay 5 µg of protein was used per well. Samples were incubated with and without delta opioid receptor agonists (10^−4^ to 10^−12^ M), for 1 hour at 25°C in assay buffer containing 30 µM GDP and 0.1 nM [^35^S]GTPγS. For whole brain membranes, the buffer used was 50 mM TrisHCl (pH 7.4), 3 mM MgCl_2_, 100 mM NaCl, 0.2 mM EGTA. In the case of spinal cord homogenates, the buffer was the same as that used for homogenization. For saturation experiments, incubation was terminated by rapid filtration and washing in ice-cold buffer (50 mM TrisHCl, 5 mM MgCl_2_, 50 mM NaCl, pH 7.4). Bound radioactivity was quantified using a liquid scintillation counter. Non-specific binding was defined as binding in the presence of 10 µM GTPγS, and basal binding indicates binding in the absence of agonist.

For Western blot analysis of phosph-DOR, DOR-eGFP mice were administered either DOR agonist (10 mg/kg) or vehicle and sacrificed 20 min later. Immediately after decapitation, the hippocampus was rapidly dissected and kept at −80°C. Frozen hippocampi were homogenized and sonicated in 2% SDS buffer containing (in mM) 50 Tris, pH 6.8, 1 EDTA, 1 sodium fluoride, and 1 sodium orthovanadate, as well as a Complete protease inhibitor mixture (Roche Applied Science). Homogenates were boiled at 96° for 4 min, and total protein content was determined by Bradford assay. Twenty micrograms of protein was loaded on a SDS-10% bisacrylamide gel and separated by constant voltage of 100 V for 1.5 h, then transferred to polyvinylidene difluoride membranes at a constant voltage of 100 V for 1 h in cold transfer buffer (Tris-borate). Membranes were blocked in 5% nonfat dry milk in a phosphate buffered saline (PBS) solution containing 0.2% Tween 20, for 2 h. Membranes were probed for phospho-DOR with primary anti-phospho-DOR (Ser363) antibody (rabbit polyclonal antibody, 1∶1000 Cell Signaling Technology) diluted in 5% bovine serum albumin and incubated overnight at 4°C. Membranes were washed three times for 10 min in PBS-0.2% Tween solution and incubated for 1 h at room temperature in horseradish peroxidase conjugated anti-rabbit IgG secondary antibodies (1∶20,000; GE Healthcare). The signal was developed using enhanced chemiluminescent reagents (ECL+; GE Healthcare) and quantified using IMAGEJ. As a loading control, β-tubulin content was analyzed on the same membrane. Membranes were stripped of antibodies for 30 min (Re-blot plus solution; Millipore), rinsed, and blocked at room temperature for 2 h. Blots were reincubated with β-tubulin primary antibody (mouse monoclonal) at room temperature for 3 hours, then washed three times for 10 min and incubated for 1 h at room temperature in horseradish-peroxidase conjugated anti-mouse IgG secondary antibodies (1∶20,000; GE Healthcare) before reaction with ECL+ solution.

To determine the subcellular distribution of DOR-eGFP after agonist stimulation, mice were anaesthetized with ketamine/xylazine (100/10 mg/kg) and intracardially perfused with 10 ml 9.25% sucrose in ddH_2_O followed by 30 ml 4% paraformaldehyde in 0.1 M phosphate buffer (PB; pH 7.4). Brains, spinal cords and dorsal root ganglia were then post-fixed for 2 hours at 4°C in the fixative solution. The tissue was then cryoprotected at 4°C in a 30% sucrose, 0.1 M PB solution until the tissue sank. Tissue was then frozen in isopentane and stored at −80°C until cut. Freely floating sections were cut at 30 µM in a cryostat. Sections were mounted on Superfrost™ glass slides in 0.01 M PBS, and DOR-eGFP receptor distribution was immediately examined in five different delta receptor-rich regions. All samples were observed under Leica confocal microscopes (SP1 or SP2UV; 63× objective and numerical aperture of 1.32), and the LCS (Leica) software was used for image acquisition. Quantification of cytoplasmic mean fluorescence intensity was determined using IMAGEJ software. Nuclear fluorescence defined the background level and was subtracted from the intracellular fluorescence measures. For each region several intracellular samples were taken and averaged to determine the intracellular mean fluorescence. In total, 5–7 different neurons were examined/region/mouse; n = 3–5 mice/group.

### Internalization in primary neuron cultures from DOR-eGFP mice

Both the preparation of primary neuron cultures, and the real time confocal microscopy were performed as described previously [13]. Briefly, P0 mice pups were decapitated, and hippocampi and striata were dissected and digested with papain (15 U/ml, Worthington). Cells were plated either on glass coverslips, or in glass-bottom dishes coated with poly-L-lysine (PLL, Sigma) in B27/NeurobasalA medium (Invitrogen) completed with 0.5 mM glutamine and antibiotics. Cells were plated at a density of 8×10^4^ cells/cm^2^. Medium was replaced 30 min after plating, and half the medium changed every 5–7 days. Cultures were maintained for 15 days *in vitro* (DIV). Fully matured primary neurons (DIV 10 to 14) were used for agonist-induced receptor internalization studies. Samples were observed under a Leica confocal microscope (SP2 AOBS MP) using a heated stage (Tempcontrol 37-2, Pecon) and 63× objective (zoom 4×) at 37°C. Images were recorded over 60 minutes, and reconstituted videos (TIMT; in-house software) contained 86 images and lasted 3 seconds. Primary neuron cultures were acutely treated with SNC80 (10 nM, 100 nM, 1 µM), or ARM390 (100 nM, 1 µM, 10 µM). When internalization occurred, it was completed by 60 min and the number of vesicles was counted manually at 30 to 46 different time points in images extracted from recorded videos. The number of DOR-eGFP vesicles at 60 min was defined as 100%. Altogether, 4 to 9 independent experiments were performed/agonist/concentration.

### Inflammatory pain in DOR-eGFP mice

All experiments were performed between 8:00–16:00 h. In all cases DOR-eGFP animals were habituated to the testing area for 20 minutes daily for 2 days prior to baseline testing. Two different variations of the Complete Freund's Adjuvant (CFA) model of inflammatory pain were used. To assess mechanical pain CFA was injected into the paw. To assess thermal pain CFA was injected into the tail. Separate groups of animals were used for each endpoint.

For mechanical responses, the threshold for responses to punctate mechanical stimuli (mechanical allodynia) was tested according to the up-and-down method [46]. In this case, the plantar surface of the animal hindpaw was stimulated with a series of eight von Frey filaments (bending force ranging from 0.01 to 2 g). Prior to the injection of CFA baseline mechanical responses (dashed line) were determined. Inflammation was induced by injecting 8 µl of CFA into the plantar surface of the paw, and animals were subsequently tested 48 hours later [27].

For thermal responses, heat hyperalgesia was assessed by immersing the tail (5 cm from the tip) into a 46°C water bath. Tail withdrawal latencies were determined, and a cut-off of 40 s was established. Prior to the injection of CFA baseline mechanical responses (dashed line) were determined. Inflammation was induced by injecting 20 µl of CFA 3 cm from the tip of the tail, and all drug tests occurred 48 hours later.

In order to ensure that all animals were treated similarly, each mouse received both i.p. and p.o. injections. Therefore, animals challenged with SNC80 (10 mg/kg, i.p.) also received a p.o. injection of dH_2_O (SNC80 group), those challenged with ARM390 (10 mg/kg, p.o.) received an i.p. injection of 0.9% saline (ARM390 group), and control animals were injected with i.p. saline and p.o. dH_2_O (Control group). Pain responses were assessed 45 minutes after drug treatment (Test 1). Mice were then re-challenged with drug or vehicle treatments 4 h or 24 h after the first test, and tested again 45 min later (Test 2).

### Statistical Analysis

All non-linear regression analysis was performed with GraphPad Prism v4 (GraphPad San Diego, CA). *In vitro* pharmacology experiments were analyzed using a one-way ANOVA. For behavioral experiments, a two-way repeated measures ANOVA was performed using Sigmastat software. Multiple comparisons were made using Bonferroni corrected t-tests. For quantification of intracellular fluorescence, a one-way ANOVA was performed, and Tukey's tests were used for post-hoc analysis.

## Supporting Information

Figure S1 SNC80 and ARM390 produce comparable pain-relieving effects. DOR-eGFP mice were tested 48 h after intraplantar injection of CFA into the paw. Separate groups of mice were challenged with differing doses of SNC80 or ARM390, and mechanical allodynia was assessed 45 min post-drug. Dashed line represents basal mechanical responses pre-CFA. *** p<0.001, two-way ANOVA, n = 3–4 mice/group.
(0.74 MB TIF)

Movie S1 Real time confocal imaging of Met-enkephalin (100 nM) induced DOR-eGFP internalization in a primary hippocampal neuron. A representative movie is shown (n = 4). Agonist was added at time 0, and remained in the bath for the full recording duration. Images were automatically recorded during 60 minutes, with increasing time intervals. For details see Methods section.
(1.83 MB MPG)

Movie S2 Real time confocal imaging of SNC80 (100 nM) induced DOR-eGFP internalization in a primary hippocampal neuron. A representative movie is shown (n = 5). Agonist was added at time 0, and remained in the bath for the full recording duration. Images were automatically recorded during 60 minutes, with increasing time intervals. For details see Methods section.
(2.55 MB MPG)

Movie S3 Real time confocal imaging of ARM390 (100 nM), which failed to induce DOR-eGFP internalization in a primary hippocampal neuron. A representative movie is shown (n = 6). Agonist was added at time 0, and remained in the bath for the full recording duration. Images were automatically recorded during 60 minutes, with increasing time intervals. For details see Methods section.
(3.12 MB MPG)

Movie S4 Real time confocal imaging of ARM390 (1 µM) induced DOR-eGFP internalization in a primary hippocampal neuron. A representative movie is shown (n = 4). Agonist was added at time 0, and remained in the bath for the full recording duration. Images were automatically recorded during 60 minutes, with increasing time intervals. For details see Methods section.
(2.18 MB MPG)
