# Supplementary figures and images for: In Vivo Delta Opioid Receptor Internalization Controls Behavioral Effects of Agonists
Source: PLoS One. 2009 May 1;4(5):e5425. doi: 10.1371/journal.pone.0005425 (PMC2672171; doi:10.1371/journal.pone.0005425)

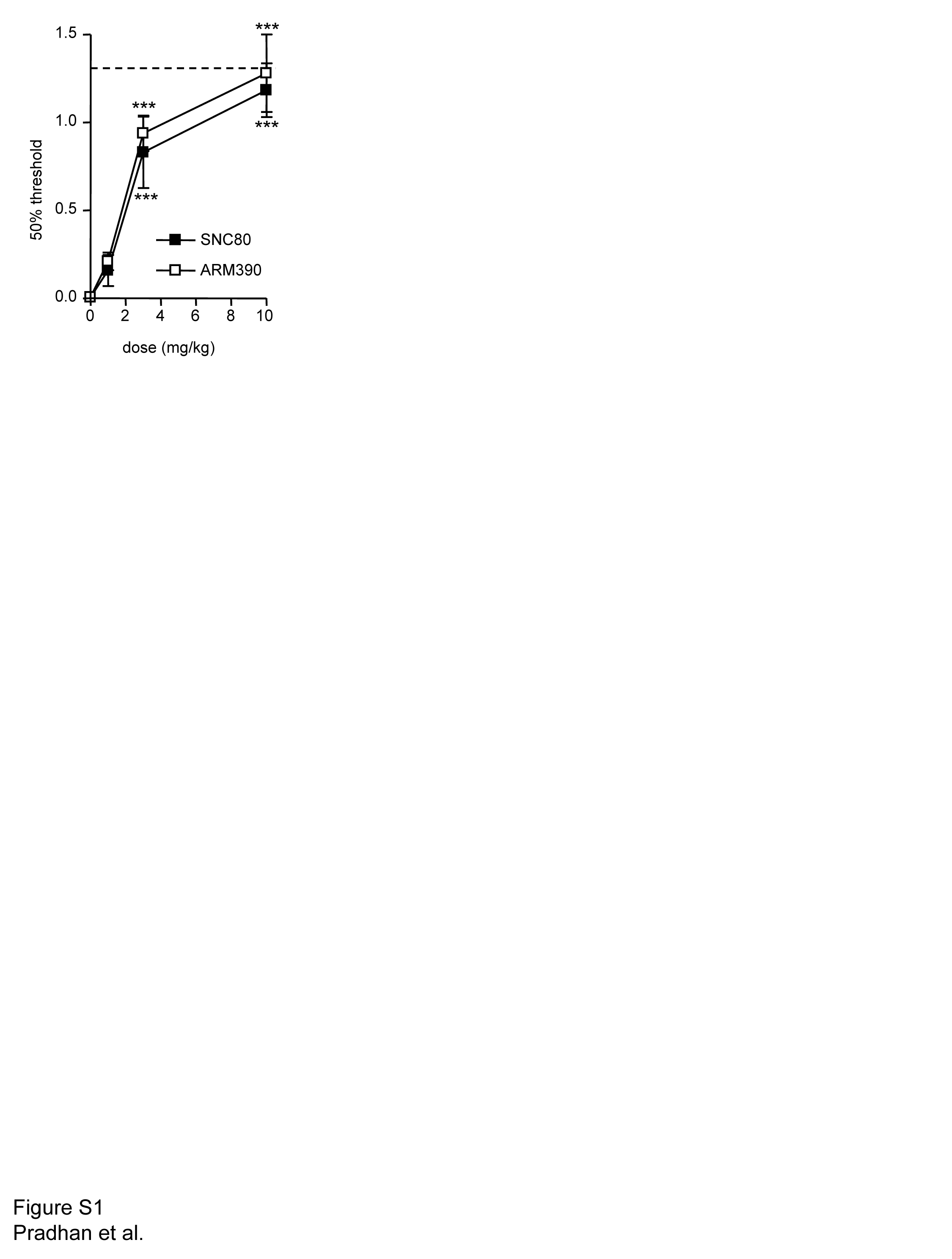

Supplement: Figure S1 — SNC80 and ARM390 produce comparable pain-relieving effects. DOR-eGFP mice were tested 48 h after intraplantar injection of CFA into the paw. Separate groups of mice were challenged with differing doses of SNC80 or ARM390, and mechanical allodynia was assessed 45 min post-drug. Dashed line represents basal mechanical responses pre-CFA. *** p<0.001, two-way ANOVA, n = 3–4 mice/group. (0.74 MB TIF) [file pone.0005425.s001.tif]
